# Clinical Implications of Androgen-Positive Triple-Negative Breast Cancer

**DOI:** 10.3390/cancers13071642

**Published:** 2021-04-01

**Authors:** Maša Brumec, Monika Sobočan, Iztok Takač, Darja Arko

**Affiliations:** 1 Department of Obstetrics and Gynecology, Faculty of Medicine, University of Maribor, 2000 Maribor, Slovenia; brumec.masa@gmail.com (M.B.); iztok.takac@ukc-mb.si (I.T.); darja.arko@ukc-mb.si (D.A.); 2 Department of Pharmacology, Faculty of Medicine, University of Maribor, 2000 Maribor, Slovenia; 3 Divison of Gynecology and Perinatology, University Medical Centre Maribor, 2000 Maribor, Slovenia

**Keywords:** androgen receptor, triple-negative breast cancer, androgen antagonist

## Abstract

**Simple Summary:**

Triple-negative breast cancer (TNBC) represents a heterogeneous group of breast cancers that lack estrogen receptor (ER), progesterone receptor (PR), and human epidermal factor 2 (HER2) amplifications. This triple negativity represents a challenge in choosing the right treatment, as without the aforementioned biomarkers there are no efficient therapeutic targets. Nevertheless, some triple-negative breast cancers express androgen receptor (AR), which could be used as a novel therapeutic target in such subgroup of breast cancers. In our review, we aimed to identify clinical features and proposed potential therapeutic approaches of this specific subgroup—AR-positive triple-negative breast cancer. Our findings contributed to a better understanding of the current problematics regarding AR-positive TNBC.

**Abstract:**

This review summarizes the recent findings of a vast array of studies conducted on androgen receptor-positive triple-negative breast cancer (AR-positive TNBC) to provide a better understanding of this specific breast cancer subgroup. AR expression is correlated with higher age, lower histological grade, lower proliferation index Ki-67, spiculated masses, and calcifications on mammography. Studies investigating the correlation between AR expression and lymph node metastasis are highly discordant. In addition, results regarding prognosis are highly contradictory. AR antagonists are a promising novel therapeutic approach in AR-positive TNBC. However, AR signaling pathways should be more investigated in order to understand the influence of AR expression on TNBC more thoroughly.

## 1. Introduction

Triple-negative breast cancer (TNBC) represents a heterogeneous group of breast cancers that are defined by absence of molecular markers. They lack estrogen receptor (ER), progesterone receptor (PR), and human epidermal growth factor receptor 2 (HER2) amplifications [1]. Expression of those markers has been proved useful for targeted therapy in other breast cancers and thus improving prognosis and outcome [2]. Unfortunately, TNBCs, therefore, lack specific molecular targets for the purpose of therapy and are consequentially associated with unfavorable prognosis [2]. Furthermore, TNBCs also differ clinically and pathologically, they express mutational and transcriptional differences, possess different genetic susceptibility, express genomic instability, and different sensitivity to chemotherapy [1]. TNBC is a heterogeneous collection of tumors with distinct phenotypes evidenced by gene expression profiling [3]. There is no standardized subtype classification of TNBC. A vast array of studies want to address the issue of heterogeneity of gene expression in TNBC and find different subtypes [3,4,5,6,7,8,9,10,11]. Lehmann et al. identified six subclasses of TNBC based on transcriptomic analysis: basal-like 1 (BL1), basal-like 2 (BL2), immunomodulatory (IM), mesenchymal-like (ML), and luminal androgen (LAR) [3]. Nevertheless, there is no standardization in the selection of biomarkers used for each subtype [12], which makes comparison of different subtypes across studies difficult. Hence, with such heterogeneity, lack of targeted therapy, reduced chemosensitivity, and poor prognosis, it is of vital importance that different biomarkers, which can help distinguish between different TNBCs and may present novel therapeutic targets, are investigated, as TNBC as such cannot be treated as one homogenous disease. One such promising biomarker and target for therapy of TNBC is the androgen receptor (AR). AR is a steroid nuclear receptor that binds testosterone and dihydrotestosterone (DHT) in the cytoplasm, and it translocates to the nucleus to regulate gene expression. It plays an important role in the cell signaling pathway, and it is essential for normal breast development and mammary cell proliferation [13,14]. It is the most widely expressed nuclear hormone receptor in breast cancer, as it is positive in approximately 70% to 90% of invasive breast carcinomas [15]. There is no association between AR expression in normal breast tissue and the subsequent incidence of breast cancer [16], but AR is typically co-expressed with ER in normal luminal cells, whereas AR downstream proteins are not expressed in AR-positive cells, regardless of ER status, age, or breast carcinoma. Furthermore, it is suggested that although AR is strongly expressed in normal luminal cells, AR signaling is not active in these cells. Nevertheless, expression status of AR may be predetermined by progenitor cell populations in normal breast tissues, and AR may exert its roles in the tumor cell proliferation, but it may not be carcinogenic by itself [17].

**Figure 1:**
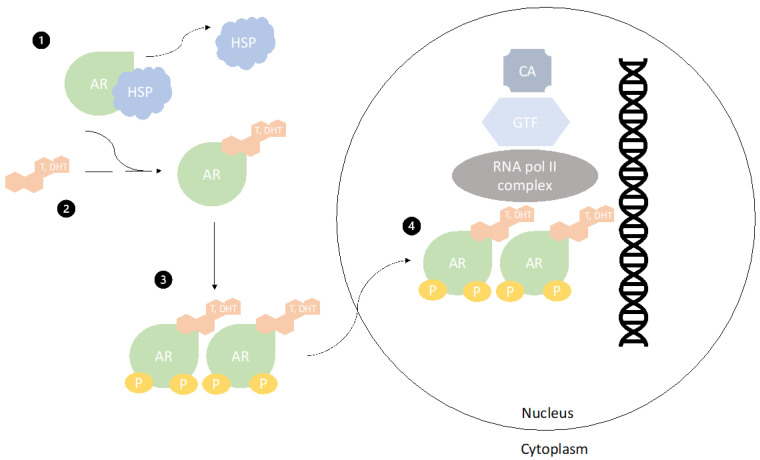
Mechanism of androgen receptor (AR) activation. Figure 1 represents how AR is activated by testosterone or dihydrotestosterone in a cell. **1**. When AR is inactive, it is bound to heat-shock proteins (HSP). **2**. Circulating androgens (e.g., testosterone and dihydrotestosterone) bind to C terminal ligand-binding domain and cause a conformational change of AR, displacing HSP. **3**. Afterwards, dimerization of AR and phosphorylation of its tyrosine kinases occurs. This change causes **4**. translocation of the complex to the nucleus where a DNA binding complex binds to androgen-responsive elements and transcription complex is formed. AR—androgen receptor HSP—heat-shock protein T; DHT—testosterone, dihydrotestosterone; P—phosphate group RNA pol II complex—RNA polymerization complex; GTF—general transcription factors; CA—coactivators.

When AR is inactive, it is bound on heat shock proteins (HSP). Most commonly in carcinogenesis, HSP70 impacts cell survival and could be a potential target for therapy [18]. They stabilize the AR in such a manner that C-terminal ligand-binding domain is exposed. Circulating androgens (e.g., testosterone and dihydrotestosterone) bind to this domain, displacing the bound HSP through conformational change. Androgen binding on AR leads to AR dimerization and phosphorylation of its tyrosine kinase, which in turn causes translocation of the AR complex to the nucleus. DNA-binding domain of AR therefore binds to androgen response elements and creates active transcription complex (Figure 1) [19]. 

Nevertheless, the role of AR in breast cancer remains unclear and therefore it is cardinal that its function is thoroughly researched. Furthermore, the interactions between the AR and androgens are complex and they differ between different sex, age, tissue type, and hormonal status [20]. AR positivity prevalence differs greatly among different studies, with most positivity rates being between 25% and 35% in TNBC [14]. The LAR subtype is especially characterized by positive AR expression and expression of luminal cytokeratins [3], but that does not implicate that only the LAR subtype is AR-positive. In addition, the LAR subtype has higher AR mRNA levels than other TNBC subtypes and expresses numerous downstream AR targets and coactivators [21]. Even though AR is expressed at a lower rate in TNBC than in other breast cancers, its presence could still be useful in diagnostic and prognostic evaluation and could provide novel insights important for managing such challenging tumor [22]. 

The aim of our review is to focus on TNBCs that express AR and illustrate the current problematics in their diagnosis, prognosis, associations with clinicopathological features, and treatment. 

## 2. Clinical Characteristics of AR-Positive Tnbc

The studies investigating associations between clinicopathological features and AR expression are highly discordant. 

Age at diagnosis was greater among the LAR subtype [3] and AR-positive TNBC [23,24,25,26,27,28,29] in comparison to other subtypes and AR-negative TNBC, respectively. This phenomenon could be attributed to the use of hormonal replacement therapy in postmenopausal patients, or to the more indolent profile of AR-positive tumors, therefore appearing in older patients [26]. However, there are studies implicating that there is no significant difference in age between different TNBC subtypes [30,31], and no significant association between AR expression and age was found [29]. The discrepancies in studies could arise due to small cohorts, as both [30,31] had less than 100 participants. 

Interestingly, a recent study [32] found significant associations between different degrees of AR expression and age. Older and postmenopausal patients had higher levels of AR in tumors than younger and premenopausal patients. AR-negative tumors were observed just as much in pre- as in postmenopausal patients. Hence, they speculated that AR-low and AR-high tumors are weakly and highly dependent on AR activity, respectively. Additionally, a very recent study hypothesized that TNBCs are entirely different entities when occurring in young or old patients, as they exhibited differences in subtypes, fibrosis, Ki-67 index, and somatic mutations [33]. Hence, knowledge about differences between TNBCs in the young and the elderly could lighten the differences in therapeutic approach, as older patients could be less responsive to conventional chemotherapy and might benefit more from a more personalized therapeutic approach (e.g., anti-androgen therapy) [33]. Even though AR-positive tumors appeared more often in postmenopausal patients [26,34,35] and a higher percentage of postmenopausal women was found in the LAR subtype [36], there was no significant correlation between AR and menopausal status found [31,37,38]. 

Among all TNBC subtypes, LAR is the most highly differentiated tumor [21]. It was found that there is an inverse relationship between AR expression and histological grade; AR expression is associated with lower histological grade [24,25,30,37,39,40,41]. On the contrary, there was no significant association between AR positivity and histological features found [31,42,43].

Ki-67 was lower among the LAR subtype than in the other subtypes [4,44,45]. Similarly, AR-positive tumors were more likely to have lower Ki-67 [28,31,34,35,46,47] which could be due to anti-proliferative effect of AR stimulation [46], suggesting a protective role or better biological fundament with expression of AR [47]. On the contrary, AR expression was not associated with Ki-67 [38]. 

There was no correlation found between AR and body mass index (BMI), but there was a strong association between AR negativity and diabetes [37]. As this is, to our knowledge, one of the few studies investigating correlation with diabetes, it is of great importance that further research is done. Additionally, not enough research is done on the associations of AR positivity and BMI, as adipose tissue is also important in the synthesis of androgens. Jongen et al. [32]. found out that the higher complete pathological response rate (pCR) was observed in younger premenopausal patients with normal BMI and lower degree of AR positivity. This observation could be attributed to chemotherapy-induced ovarian suppression, which was completely shut down as there were no local androgens synthesized by adipose tissue. In older patients with higher BMI, the tumor was not dependent on the serum androgens but on the local androgen levels from adrenal glands and adipose tissue. Therefore, patients with higher BMI should benefit less from chemotherapy since their source of tumor growth is still intact [32]. Thus, weight loss in patients with higher BMI could be of great benefit in treatment of AR-positive TNBC. It is imperative that further studies investigating the role of adipose tissue in TNBC patients are conducted, as they are significantly lacking. 

## 3. Potential Diagnostic and Prognostic Biomarkers

### 3.1. SOX10 and GATA3

Breast cancer can metastasize to almost any organ. Since differentiation of carcinomas of unknown primary origin can be a challenge for a clinician, it is of great importance that novel biomarkers for diagnosis of primary breast cancer are found [48].

*SOX10* plays a role in mediated mammary epithelial cell growth in vitro and it labels benign myoepithelial cells of the breast, as well as myoepithelial cell-derived neoplasms of the salivary glands. Therefore, it is suggested that it could be attributable to a basal-like or myoepithelial cell-like phenotype of these neoplasms, and it could be a potential biomarker for TNBCs. In relation to the AR, the incidence of AR expression is lower in *SOX10*-positive TNBC than in *SOX10*-negative TNBC [49,50]. In other words, *SOX10* and AR expression are inversely correlated. Taken together, AR is expressed in many breast carcinomas, but cannot be used as an independent biomarker, whereas *SOX10* could be [50]. Even though its significance in diagnosis of AR-positive TNBC is limited due to the inverse relationship between *SOX10* and AR, it could still be used for diagnosis of AR-negative TNBC, the so-called quadruple-negative breast cancer (QNBC). Furthermore, *SOX10* in conjunction with *GATA3*, could be used as additional diagnostic marker of metastasis of unknown origin [49,50]. Additionally, *SOX10* is useful because its expression is stable between primary and metastatic lesions and exhibits the highest level of concordance in comparison with *GATA3* and AR [14]. 

*GATA3* is a zinc finger transcription factor, and it is a key regulator of cellular differentiation and lineage specification and has different roles in the human body. In mammary gland, it is responsible for differentiation of luminal cells and low *GATA3* expression correlates with worse breast cancer prognosis [51]. *GATA3* expression was strongly correlated with AR positivity, suggesting a pathway linking *GATA3* and AR signaling [52,53]. Specifically, correlation of *GATA3* and AR was stronger in lobular carcinomas, suggesting that the pathways linking them are more active in tumors with lobular differentiation. Furthermore, *GATA3* expression was negatively associated with nuclear grade, which suggests that it is a marker of better differentiation [53]. Additionally, the observed results are consistent with the finding that AR expression is associated with lower histological grade. 

The significance of *GATA3* staining is proved useful, especially in differentiation between breast and urothelial carcinoma, as they share overlapping morphology [48]. (DAVIS) *GATA3* and AR correlation can be used in *GATA3*-positive tumors of unknown origin to distinguish metastatic *GATA3*-positive carcinoma of breast origin from urothelial origin [53].

On the contrary, the expression of AR was not associated with *GATA3* expression [54], but the discrepancy in the results could be attributed to a small sample of the latter study.

Taken together, both *SOX10* and *GATA3* are useful in distinguishing between primary tumor and metastatic breast cancer [14], the former is more appropriate for AR-negative tumors and the latter for AR-positive tumors. 

### 3.2. Programmed Cell Death Ligand (PDL1) and Forkhead Box 1 (FOXA1)

Recent scientific research is emerging to identify novel prognostic biomarkers for targeted analysis and therapy of TNBCs [55]. PD1 is an immune checkpoint receptor and a cardinal immunosuppression mechanism used by cancer cells to escape host immunity [55]. Its ligand, PDL1 predicted AR positivity, as AR-positive TNBC was threefold more likely to express PD-L1 on cancer cells. If confirmed, combination therapy with AR inhibitors and immune therapy could be an appropriate therapeutic approach for such tumors [27]. On the contrary, in other studies, PDL1 expression on tumor cells [56] and on TILs was not significantly different between AR-negative and AR-positive tumors [57], but a higher rate was observed in the AR-positive and FOXA1-negative subgroups [56]. Furthermore, among patients with PD-L1-positive tumor, poorer relapse-free survival (RFS) and overall survival (OS) were observed in the case of co-expression of AR and FOXA1. Therefore, immunotherapy with PDL-1 targeted therapies could be appropriate for this cohort of patients [56].

FOXA1 is a key determinant of ER function and endocrine response. There is a significant association between AR and FOXA1 status [55,58]. In one study, co-expression of AR and FOXA1 was found in 15% of all TNBCs [26] and it was associated with shorter DFS [55]. Additionally, AR-positive and FOXA1-positive subgroups have shown higher rates of PIK3CA mutations and, therefore, a concomitant evaluation of this subgroup is suggested, as they could probably benefit from PIK3CA inhibitors, alone or in combination with antiandrogens [56]. FOXA1 and AR co-expression should be further researched, as it may identify a specific subgroup of TNBC.

### 3.3. ER-Beta Receptor

ER-beta receptor is the other form of ER and it has been, unlike ER-alfa receptor, detected in TNBC patients [59], more frequently in AR-positive TNBC [60]. ER-beta receptor overexpression upregulated PTEN and decreased the phosphorylation of AKT and downregulated AR expression in MDA-MB 453 cells. Due to non-genomic anti-proliferative effects of ER-beta, it could be a novel predictor for better clinical outcomes in AR-positive TNBC [59]. Another study was conducted by Song et al., supporting the latter results, as they found out that ER-beta2 inhibited migration and reduced the invasiveness of AR-positive TNBC, thus suggesting the ER-beta as a potential prognostic marker [60].

## 4. Clinical Imaging and AR Positivity

The growth pattern of a tumour also contributes to identifying appropriate therapeutic regimen for TNBC patients along with the pathological characteristics. Even though the differences are not so significant between TNBC and non-TNBC patients, it is believed that there are differences among different histological subtypes [36]. Studies showed that both in LAR and other AR-positive TNBC, the lesions appear more frequently as mammographic calcifications or as spiculated masses [36,42,43,58,61]. Furthermore, it is speculated that this is due to lower percentage of poorly differentiated tumors, as they are more prone to have infiltrating growth pattern, between LAR-TNBC [62]. Additionally, the irregular shape suggest that AR-positive tumors are less proliferative and hence it is not surprising that they exhibit lower Ki-67 expression [43]. 

Due to its distinct growth pattern, high rate of missed malignant diagnoses between different TNBC subtypes was observed. The lowest rate of misdiagnosis in mammography was observed among the LAR subtype [63]. 

However, it was found that AR negativity was associated with higher density on mammography among clinically detected tumors, whereas there was no association found among screening-detected cases [64].

## 5. Prognosis

The influence of AR expression on prognosis is still being controversial, as the role of AR signaling in TNBC tumor cells is still not well understood.

It was found that prognosis highly depends on the ER status. AR expression is associated with a better prognosis in patients that have ER-positive tumors, whereas ER-negative tumors do not display such a pattern [15]. It was postulated that AR acts in an antiproliferative manner in ER-positive tumors by antagonizing ER, whereas it facilitates tumor cell growth in ER-negative tumors in an androgen-dependent manner [65].

AR positivity is associated with greater mortality in TNBC patients [15]. Furthermore, a poor DFS was observed in AR-positive TNBC and hence supporting the hypothesis that a pharmacological AR block is a potential therapy [55]. Similarly, shorter overall survival (OS) and disease-free survival (DFS) values were observed in AR-positive tumors [25]. Additionally, a significant association was found between AR and FOXA1, as a shorter DFS for AR-positive and FOXA1-positive TNBC tumors was reported [55]. Shorter DFS was also found in BRCA1-negative and AR-positive TNBC patients.

On the contrary, Thike et al. reported that women with AR-positive TNBC had significantly improved DFS and better OS. Additionally, these tumors have a decreased likelihood of recurrence, whereas AR-negative tumors are associated with development of recurrences and prone to develop metastases [38,39,40,66]. Similarly, it was found out that AR-negative tumors were significantly associated with aggressive behavior and a shorter OS [67,68]. 

AR immunohistochemistry (IHC) expression was statistically associated with better survival, even though it had a statistical association with LNM metastases [37]. Lymph node metastasis was significantly more frequent in the LAR subtype, and there was a statistical association between AR-positive TNBC and lymph node metastases [37,41,63,69]. On the contrary, some studies found no association between AR expression and lymph node metastasis [26,30,38].

Additionally, it was demonstrated that AR-positivity was significantly correlated with better survival in specific high-risk subgroups: young, premenopausal, large tumor size, more node involvement (4+), high stage, high grade, positive vascular invasion, positive p53, negative CK5/6, and higher Ki-67. Thus, it was suggested that expression of AR reveals tumors with better biological behavior in high-risk patients [34]. Some studies also showed no significant difference in survival among AR-negative and AR-positive tumors [15,30,31,70], but still AR expression was useful as a prognostic factor in advanced stage tumors [70]. 

Distant-metastasis-free survival did not vary between different TNBC subtypes, whereas relapse-free survival (RFS) was increased in the LAR subtype, which suggests that the recurrence was due to different mechanisms and not connected to AR [3]. 

### 5.1. AR as an Independent Prognostic Marker

AR was demonstrated to be an independent prognostic marker for both DFS and OS [34]. On the contrary, Rakha et al. found that AR expression was not observed to be an independent prognostic marker in TNBC patients. However, AR status was useful in determining prognosis of lymph node-positive tumors as a negative AR expression was associated with higher histological grade [39]. AR is not a good biomarker for the existing subtypes of breast cancer, even though its expression could be used to define subtype stratification [50]. Furthermore, Mansouri et al. supported the idea that AR expression on its own cannot be an independent prognostic marker, whereas the co-expression of AR and Cathepsin-D could be an independent prognostic factor for OS. Cathepsin-D is a lysosomal endoproteinase that is proteolytically active at low pH and is overproduced and hypersecreted by breast cancer cells [57]. Similarly, it was found that AR expression alone cannot be an independent prognostic marker, whereas co-expression of AR and FOXA1 was [56]. Additionally, AR-positive and FOXA1-positive phenotype represented a specific subgroup of patients with poor prognosis [55,56]. Therefore, the co-expression of different biomarkers and AR should be considered when searching for a prognostic factor. 

### 5.2. Discordance Rate of AR Positivity

A high discordance rate is present in biomarker expression between primary and metastatic lesions [14,24], as well as between primary tumor and recurrent lesion [66]. Likewise, a significant degree of discordance in biomarkers among all breast cancer subtypes has been studied, with the highest discordance rate among TNBC [23]. When AR was negative, TNBC discordance rate between primary, metastatic, and recurrent tumor decreased. Data suggest that AR-positive TNBC are even more heterogeneous than previously thought. However, Gasparini et al. showed no significant difference in AR expression between primary and metastatic lesion. Similarly, McNamara et al. showed that AR status was preserved in both metastatic and recurrent tumors [47]. Interestingly, it was found that there was an increased AR status in bone metastasis and a decreased status in brain metastasis [47], which is of great interest, as TNBCs usually metastases toward brain, and association between AR expression and brain metastasis should be further evaluated [71]. It is suggested that AR loss could be associated with the metastatic process and suggested further evaluation of the discordance rate [72]. 

## 6. Androgen Receptors as a Therapeutic Target

Since the role of AR in involvement of breast cancer is unclear, it has been contentious whether AR agonists or antagonists should be used in the treatment. It was discovered that AR agonists can inhibit proliferation of AR-positive breast cancer, except in ER-negative and HER2-positive breast cancer [73]. Hence, the effects of both AR agonists and antagonists were tested on AR-positive and AR-negative cell lines. AR agonists DHT and R1881 showed anti-proliferative effects in TNBC and the response was AR-specific. Conversely, a decrease in cell proliferation was observed by application of AR antagonists regardless of their AR protein expression, suggesting that AR antagonists are AR-independent. These results suggest that the anti-proliferative effect of AR antagonists could be due to an off-target effect and, therefore, further genetic background should be investigated. The off-target AR-antagonist effect was not observed in prostate cells, and it is suggested that there is tissue-specific regulation of signalling pathways present [73]. Hormone dependant modulation of breast cancer has long been hypothesized to be a viable route of treatment [74]. Recent data shows that there is a synergistic effect of AR agonists with cyclin-dependent kinases (CDK), CDK4, and CDK6. Conversely however, selective ER modulator tamoxifen did not show a synergistic effect with AR agonists [75]. The conclusions of recently published data suggest that in ER-positive breast cancer, in order to induce and enhance anti-tumor effects, AR agonist should be used and in ER-negative breast cancer, more focus should be put on treatment with ER antagonists [74,75,76]. 

In multiple non-LAR breast cancer subtypes, relatively low AR expression depended on AR for proliferation, migration, and invasion [77]. Furthermore, the sensitivity of cancer cells to immune-mediated lysis was independent of detectable AR expression, both in enzalutamide and abiraterone treatment. AR expression was not required for the immunomodulatory effects of enzalutamide [78], but inhibition of cell migration and invasion exhibited by enzalutamide was AR-dependent [79].

Bicalutamide, a first-generation AR antagonist, was shown to induce cell apoptosis [80] and inhibit cell motility and invasiveness in cell line MDA-MB-453 [81]. Using cell lines that represent the LAR subtype, Lehmann et al. showed that they are sensitive to AR antagonist bicalutamide and 17-DMAG [3]. 

Enzalutamide, a second-generation AR antagonist, which is a competitive inhibitor that prevents AR nuclear localization [82], was shown to have clinical activity in treatment of AR-positive TNBC [83]. Furthermore, it has higher anti-proliferative effects than the first-generation AR antagonists [59,84], as it exhibits a higher affinity for AR, represses AR nuclear translocation, and decreases DNA binding and activation of coactivators [84]. Additionally, the expression of ER-beta in MDA-MB 453 cells increased the sensitivity of cells to enzalutamide. This finding poses additional questions about whether ER-beta expression should also be evaluated in ongoing clinical trials studying enzalutamide, as it may hold a predictive role for endocrine responses to anti-androgens in AR-positive TNBC cells. The mechanism behind this phenomenon could be that the AR enters the nucleus as a heterodimer with ER-beta and therefore does not bind to androgen-responsive elements to promote cell growth [59]. 

More than that, AR inhibition with enzalutamide was shown to be an inductor of radiation sensitivity in AR-positive TNBC cell lines, proposing AR inhibition as an effective radiosensitization strategy [85]. Additionally, seviteronel, a CYP17 lyase inhibitor, which therefore inhibits synthesis of androgens and estrogens [86] and also acts as a competitive antagonist of AR [87], has showed a unique mechanism of radiosensitization compared to enzalutamide [88].

The large-conductance Ca^2+^-activated K^+^ channel K_Ca_1.1. plays an important role in the promotion of breast cancer cell proliferation and metastasis. It was also found to be an androgen-responsive gene in AR-positive breast cancer. Anti-androgens also affect K_Ca_1.1. in AR-positive breast cancer cells and its down-regulation may contribute at least in part to the anti-proliferative and anti-metastatic effects of androgen receptor inhibitors. The mechanism behind anti-androgen actions in the inhibition of K_Ca_1.1. activity is thought to be the enhancement of K_Ca_1.1. protein degradation [89].

Abiraterone inhibits CYP17A1—an enzyme involved in androgen biosynthesis. It leads to reduced AR signaling, as there is a decreased amount of androgens required to stimulate the signaling cascade [90]. It was shown that TNBC tumors with apocrine features showed more clinical benefit when treated with abiraterone. Furthermore, it was demonstrated, by using differentially expressed genes in responders and non-responders, that Chk1 inhibition improves abiraterone efficacy in vitro and in vivo. Chk1 is a protein kinase that is essential for maintenance of genomic integrity. It was also shown that non-responders to abiraterone showed an overexpression of CHEK1, a gene encoding Chk1 [91].

### Interplay of Signaling Pathways with Androgen Receptors

The phosphatidylinositol 3-kinase (PI3K) signaling pathway is vital for cell growth and survival [21], and PIK3CA mutations are more abundant in LAR subtype [92,93], which is consistent with the finding that they are also more frequent in elderly patients [33]. Mutations associated with expression of AR [93,94] and LAR TNBC were significantly enriched with PIK3CA and AKT1 mutations [21], suggesting an important role of this pathway in communicating with AR. Inhibition of PI3K pathway with BEZ235 (PI3K-mTOR inhibitor) decreased the amount of AR in the absence of androgens. It also showed a positive correlation between expression of EGFR, PDGFR-beta, and the expression of AR. Also, an additive anti-proliferative effect of EGFR, PDFGR-beta, and Erk1/2 inhibition (lapatinib, imatinib mesylate and PD98059, respectively) was observed even more when given with bicalutamide. These findings suggest that the administration of anti-androgens should be given with inhibitors of PI3KCA or MAPK signaling [95]. A combination of AR antagonist and PI3K/mTOR inhibitor may show synergistic results [3]. This is also supported by another study investigating a combination of enzalutamide and PI3K inhibitor taselisib. The combination exhibited significant clinical benefit compared to no clinical benefit when treating with enzalutamide alone. In this study, the LAR subtype exhibited higher clinical benefit in comparison to other TNBC subtypes. Thus, in addition to expressing AR protein, the presence of LAR gene signature may identify patients most likely to benefit from AR antagonists [96]. Similarly, it was showed that the growth and viability of AR-positive TNBC cell line models were reduced after dual treatment with AR antagonists and PIK3CA inhibitors [94]. High expression of p-mTOR may drive tumor proliferation in almost one third of TNBC, and the p-mTOR positivity is associated with AR expression. These results suggest that there may be a subgroup of TNBC patients which could benefit from synergistic effects of AR inhibitors and mTOR inhibitors [97]. The mTOR inhibitor rapamycin and the anti-androgen enzalutamide had additive effects also in the cell line MDA-MB-453 cells [98]. In addition, an important therapeutic method for LAR subgroup of TNBC [99,100], the CDK 4/6 inhibition (palbociclib) is clinically emerging in the last years. It was shown that substantial activity impact of CDK4/6 and PI3K inhibitor combination in PIK3CA TNBC is present, indicating that this subgroup of patients could benefit from CDK4/6 inhibitors [101]. 

## 7. Discussion

Recently, a lot of interest has been put into investigation of TNBC, as it represents a highly heterogeneous and challenging disease. Our review shows that age at diagnosis is higher in AR subtype breast cancer and AR-positive TNBC (Table 1—clinicopathological differences) [26]. Nevertheless, it was not shown that there is a significant difference in age between different subtypes, but this could also be due to small sample size [30,31]. Discourse currently leads to the belief that TNBCs occurring in younger and older patients are two different entities [33]. Hence, knowledge about differences between different TNBCs in the young and the elderly, could lighten the differences in therapeutic approach, as older patients could be less responsive to conventional chemotherapy and might benefit more from a more personalized therapeutic approach. Even though AR-positive tumors and LAR tumors appeared more often in postmenopausal patients, there was no significant correlation between AR and menopausal status found [31,37,38]. 

Regarding lifestyle impact, we encountered only a few studies investigating AR expression in correlation to BMI and/or diabetes. As diabetes and higher BMI are associated with higher age and adipose tissue is an important part of androgen synthesis, we believe that it is of vital importance that more research is conducted in this particular area. Furthermore, the role of weight loss in treatment of AR-positive TNBC should also be investigated. 

There is still a vivid discussion regarding the AR positivity cut-offs, since they vary among studies. Most studies report either 1% or 10% of AR-positive cells present in a tumor sample as positive [26,75]. If classifying patients into three categories: negative (<1%), 1–34% (AR-low), and >34% (AR-high), one can find significant differences among subgroups leading to believe it is dependent on different dependency on AR activity [32]. But AR positivity is not the only determinant for tumor androgen pathway activity. AR can still be activated via one of its many phosphorylation sites. Hence, AR phosphorylation rather than total AR expression would present new population of tumors appropriate for anti-androgen therapy [103,104]. A significant proportion of tumors (e.g., molecular apocrine) may express AR mRNA via qRT-PCR, even though they are AR-negative via IHC. Consequentially, IHC-based detection of AR expression may not be sensitive enough to identify all TNBC tumors in which AR-mediated signaling is active [105]. It is questionable which method of molecular classification to determine AR positivity is the most appropriate. 

The clinico-pathological features show that studies on histological grade were in agreement, showing that AR-positive tumors and LAR tumors are highly differentiated and thus have lower histological grade. It was observed that during tumor progression, a change of AR expression is present. This could be due to genetic drifts during tumor progression. Secondly, it could be explained due to limited accuracy and reproducibility of receptor assays or different tissue handling, processing, interpretation of the results, different cut-off values used for AR positivity etc. Thirdly, intratumoral heterogeneity of the primary tumor also plays an important role [24]. Due to high discordance rate in the breast cancer continuum, it is very important that biopsy with molecular retesting is performed, at both initial diagnosis and every recurrence, in order to avoid inadequate treatment for patients [23].

Additionally, biological roles of androgens in TNBC are controversial and therefore, further research on involvement of androgen signaling pathways should be conducted and novel markers indicating androgen activation should be found.

TNBC is a very challenging disease to treat due to lack of molecular targets and with identification of new molecular biomarkers, such as AR, a novel therapeutic approach has been proposed. In addition, AR-positive TNBC are more challenging, as they have a low Ki-67 index and are therefore less responsive to chemotherapy. Furthermore, acquired chemoresistance and change in biomarker expression was also observed. Major controversies regarding the influence of AR expression on the prognosis are present. Firstly, the discrepancies between the studies could be because the patients received inappropriate treatment due to a lack of targeted treatment for AR-positive TNBC and thus the results between the studies are hardly comparable. Secondly, as it is observed that AR-positive TNBC is more common in older patients, it could be that the treatment in many cases was not possible due to age-related comorbidities [3]. We found out that AR antagonists bicalutamide and enzalutamide are widely researched and have been shown to be effective. Their effectiveness was even higher in combination with other drugs, e.g., EGFR inhibitor, PDFGR-beta inhibitor, Erk1/2 inhibitor, PI3K inhibitors, mTOR inhibitors, PARP1 inhibitors, CDK4/6 inhibitors, BET inhibitors. A combination of CDK4/6 inhibitors and AR antagonists proved even more effective in patients that were AR- and RB-positive, expanding the subgroup of patients that could benefit from CDK4/6 inhibitors. There are a lot of novel potential therapeutic targets such as SARMs, ARNILA, BRD and BET inhibitors etc. Additionally, PI3K and mTOR inhibitors were shown to be successful in LAR TNBC resistant to enzalutamide. Nevertheless, we still do not know precisely why AR antagonists are effective and their effectiveness could be due to an off-target effect. Hence, it is very important that AR signaling pathways are further studied, and regulation mechanism of AR pathway should be understood for better therapeutic approach [106]. 

## 8. Conclusions

TNBC is a highly heterogeneous disease that lacks specific therapeutic targets. Androgen receptor expression is correlated with higher age at diagnosis, lower histological grade, lower proliferation index Ki-67, and specific mammographic results (spiculated masses and calcifications). Despite enormous amount of knowledge gathered on TNBC to date, the data regarding prognosis are still contradictory. Research has shown that AR could be a promising therapeutic target. However, AR signaling pathways are not fully understood and more complicated than thought, as AR also interplays with other signaling pathways, especially PIK3CA and MAPK pathways. In conclusion, we recommend that more personalized approach is taken in treatment of TNBC. 

## Figures and Tables

**Table 1:** Table is representing major studies investigating clinical-pathological features of AR-positive triple-negative breast cancer (TNBC) and luminal androgen (LAR) TNBC.

First Author	Type of Study	*n*	AR threshold for Positivity	Method of AR Assessment	% of AR-Positive/LAR Tumors	Clinical Features (Only Results Relevant to Our Review are Presented)
Lehmann et al. [3]	Analysis of breast cancer data sets	587	No value applied	Gene expression analysis	11% LAR	Patients in the LAR group were significantly older at diagnosis.
Kim et al. [24]	Retrospective study	55	Allred scoring method	IHC	14.5% AR-positive	AR expression in TNBC was associated with older age at diagnosis (*p* = 0.006), smaller tumor size (*p* = 0.032) and lower histologic grade (*p* = 0.003).
Choi et al. [25]	Retrospective study	492	1%	IHC	17.7% AR-positive	AR expression showed significant correlation with older age (*p* < 0.001), lower histologic grade (*p* < 0.001). Poor prognostic marker for OS in univariate (*p* = 0.026) and multivariate (*p* = 0.008) analysis.
Guiu et al. [26]	Prospective study	592	10%	IHC	26%	AR-positive tumors had lower nuclear grades, appeared more often in older and postmenopausal women, exhibited less often lymphocytic infiltrate. No association between AR expression and tumor size, node involvement.
Tung et al. [27]	Retrospective study	197	Negative: <1% Weakly positive: 1–10% Positive: >10%	IHC	18%	AR expression was less common in *BRCA1*. Factors predicting AR expression were: lower histologic grade, older age at diagnosis and PDL-1 expression.
Dieci et al. [28]	Retrospective study	263	1%	IHC	29.7%	AR expression was presented more frequently with older age (*p* > 0.001), G1-G2 (*p* = 0.003), lower Ki-67 (*p* < 0.001).
Astvatsaturyan et al. [29]	Retrospective study	135	1%, 10%, 20%, 25%, 30%	IHC	41% at 1% threshold	AR immunoreactivity in at least 1% of tumor cell nuclei was considered the most appropriate threshold to define AR positivity. Using this threshold AR-positive tumors were more frequently in older women, inverse relationship between Ki-67 and AR expression was found.
Sunar et al. [30]	Retrospective study	84	1%	IHC	29.8%	No statistically significant differences in terms of age, tumor size, lymph node metastasis. Grade 3 tumors were less frequent in AR-positive tumors.
Pistelli et al. [31]	Retrospective study	81	10%	IHC	18.8%	AR expression was inversely correlated with a higher Ki-67 and a lymphovascular invasion, but no other variables (age, menopausal status, size of tumor, histological features).
Park et al. [102]	Retrospective study	413	10%	IHC	35%	AR was significantly expressed in patients with smaller tumor size (*p* = 0.035) and lower histologic grade (*p* < 0.001). There were no statistically significant differences between AR expression and age at diagnosis, BMI, menopausal status, lymph node involvement.
Hu et al. [34]	Retrospective study	360	10%	IHC	31.4%	AR-positive tumors were more likely to have low Ki-67 (*p* = 0.007), observed in post-menopausal patients (*p* = 0.037), grade 3 (*p* = 0.007). AR expression was not correlated to patient age, tumor size, node status and vascular invasion.
Jongen et al. [32]	Retrospective study	71	AR-high: >34% AR-low: 1–34% >1%, >10%, <1%	IHC	1% cut-off: 32% 10% cut-off: 27% Degrees: 15% AR-low 17% AR-high	Younger and premenopausal patients carried more AR-low tumors, AR-high tumors observed more frequent in older and postmenopausal patients. AR-negative in the middle regarding age.
Mohammed et al. [35]	Retrospective study	89	1%	IHC	32.6%	Ki-67 and histological grade were lower in AR-positive group. No significant association was observed between pCR and clinical-pathological features in AR-positive TNBC.
Mueller et al. [36]	Retrospective study	135	Not applicable	Subtyping using a panel of antibodies	27.4% were LAR TNBC	Mammographic margins of LAR TNBC more often spiculated or presented as a mass with calcifications.
Collina et al. [37]	Retrospective study	238	1%	IHC and expression of luminal cytokeratin for identification of LAR	LAR: 19% AR: 23%	AR expression was not correlated to menopausal condition, both in AR-positive and LAR TNBC patients. No correlation was also found with BMI, but a strong association between AR downregulation and diabetes was found.
Asano et al. [38]	Retrospective study	190	1%	IHC	29.5%	No correlation was found between clinicopathological characteristics and AR expression.
Rakha et al. [39]	Retrospective study	1944 (of which 282 were TNBC)	0% (negative)	IHC	23% of TNBC cases	Absence of AR was associated with higher histological grade (*p* < 0.001), development of recurrences (*p* = 0.038) and distant metastasis (*p* = 0.049).
Thike et al. [40]	Retrospective study	699	1%	IHC	38% AR-positive	Androgen receptor expression was inversely correlated with histologic grade and mitotic score.
Shen et al. [41]	Retrospective study	165	10%	IHC	35.8% high levels of AR and 64.2% low levels of AR	Expression of AR was positively associated with tumor size, lymph node metastasis and high-grade tumor.
Bae et al. [42]	Retrospective study	125	10%	IHC	26.4% AR-positive	AR-positive TNBC is associated with calcifications, spiculated masses, and non-mass enhancement.
Candelaria et al. [43]	Prospective study	144	10%	IHC	31.2% AR-positive	AR-positive TNBC was significantly associated with heterogeneously dense breast composition on mammography, mass with calcifications, irregular mass shape on mammography, and irregular mass shape on sonography.
Kim et al. [44]	Retrospective study	200	1%	IHC	11% LAR	LAR subtype was associated with older patient age, apocrine histological features, low density of stromal tumor-infiltrating lymphocytes and low Ki-67 labeling index.
Santonja et al. [45]	retrospective study	125	1%	IHC	11.2%	LAR is the least proliferative subtype and the most chemoresistant one.
McNamara et al. [47]	Retrospective study	39	H score	IHC	Data not shown	AR status was concordant between primary and recurrent/metastatic disease, but coordinated expression of AR and androgenic enzymes was lost. There was an inverse association between AR and Ki-67.
Elfgen et al. [63]	Observational study	166	Semi-quantitative scoring system	IHC	28.3% LAR	Lowest rate of missed malignant diagnoses on mammography was found in LAR. Lymph node metastasis was significantly more frequent in LAR.
Luo et al. [69]	Retrospective study	137 TNBC and 132 non-TNBC	1% (different scores were given to the percentage of positive cells and staining intensity)	IHC	27.7% in TNBC and 83.3% in non-TNBC	Positive rate of AR was significantly lower in TNBC than non-TNBC. AR expression was correlated with menorrheal status (*p* = 0.009), tumor grade (*p* = 0.023), node status (*p* = 0.005), but was not correlated with clinicopathologic parameters and survival in non-TNBC.
